# Association of Gene Polymorphisms in APOE and BIN1 With Dementia of Alzheimer's Type Susceptibility in Chinese Han Population

**DOI:** 10.3389/fpsyt.2021.753909

**Published:** 2021-10-18

**Authors:** Xiaoyue Li, Yelei Zhang, Xinyu Chen, Hongwei Yuan, Zhiqiang Wang, Guoqiang Wang, Kai Zhang, Huanzhong Liu

**Affiliations:** ^1^Department of Psychiatry, Chaohu Hospital, Anhui Medical University, Hefei, China; ^2^Anhui Psychiatric Center, Anhui Medical University, Hefei, China; ^3^Department of Psychiatry, Wuxi Mental Health Center, Nanjing Medical University, Wuxi, China

**Keywords:** dementia with Alzheimer's type, APOE, BIN1 bridging integrator 1/amphiphysin-2 gene, single nucleotide polymorphism, genetic associations

## Abstract

**Objectives:** Dementia of the Alzheimer's type (DAT) is the most common chronic neurodegenerative disease. At present, the pathogenesis of DAT is not completely clear, and there are no drugs that can cure the disease. Once an individual is diagnosed with DAT, the survival time is only 3 to 9 years. Therefore, there is an urgent need to determine the etiology of DAT and the associated influencing factors to find a breakthrough in the treatment of DAT.

**Methods:** We studied the relationship between polymorphisms in several genes (including BIN1 and APOE) and DAT susceptibility and the effects of sex differences on DAT. Our study included 137 patients with DAT and 509 healthy controls (HCs).

**Results:** The APOE rs429358 polymorphism CC and CT genotypes were associated with an increased risk of DAT in women. We found a significant association between APOE ε4 and DAT. The frequency of the ε4 allele in the DAT group (15.5%) was higher than that in the HC group (8.7%). The BIN1 rs7561528 polymorphism was associated with a decreased risk of DAT in men.

**Conclusions:** APOE gene rs429358 and BIN1 gene 7561528 genes may affect the susceptibility to DAT in a Chinese Han population.

## Introduction

Dementia of the Alzheimer's type (DAT) is the most common chronic neurodegenerative disease, affecting 44 million people worldwide, and is the fifth leading cause of death (1). The prevalence of dementia in China reached 9.19 million in 2010, with an average of 6.25 individuals per thousand people suffering from DAT (2). Although the rate of DAT progression varies, once diagnosed, the survival time of a patient with DAT is only 3–9 years (3). DAT is characterized by amyloid deposition in the hippocampus, early brain oxidative stress, and the development of nerve fiber tangles caused by the hyperphosphorylation of tau protein (4). The result of these pathological changes is a series of clinical features, such as learning and memory impairment, progressive aphasia, and decreased executive function (5). Genetic variants have been reported to seriously affect DAT susceptibility, with many genes, including BIN1, APOE, PICALM, and probably many more, being involved (6). Therefore, there is an urgent need to determine the etiology of DAT and the associated influencing factors to find a breakthrough in DAT treatment. Further understanding of DAT genetic susceptibility may be helpful for individual risk assessment and early intervention.

Apolipoprotein E (APOE) is a protein composed of 299 amino acids that is produced by hepatocytes, adipocytes, and other cells. APOE mainly transports lipids in the plasma and nervous system. In addition, APOE is involved in regulating the integrity and plasticity of synaptic proteins (7). APOE plays an important role in many diseases, including neurodegenerative diseases such as depression, dementia with Lewy bodies (DLB), Parkinson's disease (PD), and multiple sclerosis (MS). APOE has been reported to be associated with susceptibility to depression, and the APOE ε2 allele may be an important protective factor for depression (8). APOE participates in and promotes the development of DLB by directly participating in pathological changes in Lewy bodies and the maintenance of lipid homeostasis in the brain (9). In addition, the levels of APOE protein and its receptor in the plasma of patients with PD were found to be significantly increased. APOE ε4 is a risk factor for early-onset and cognitive impairment in PD (10). Finally, in patients with MS, the APOE allele is associated with poor speech learning task performance, persistent attention loss, and cortical impairment (11, 12).

At present, it is generally believed that the APOE gene is involved in the occurrence and development of DAT (13, 14). As a glycoprotein, APOE is a risk factor for DAT by participating in the inflammatory response of the nervous system and maintaining lipid homeostasis in the brain (15). The level of APOE in cerebrospinal fluid has been shown to be positively correlated with the level of phosphorylated tau protein. Therefore, the level of APOE may be a potential biological indicator of DAT (16). Animal experiments and epidemiological studies have confirmed that apolipoprotein E gene polymorphisms are associated with DAT (17, 18). APOE encodes three alleles: APOE ε2, APOE ε3 and APOE ε4. APOE ε2 is generally considered to be a protective factor for DAT, while APOE ε4 is the most high-risk genetic predictive factor known (19, 20). Therefore, we evaluated the impact of rs429258 and rs7412 on the risk of DAT in the Chinese population.

Bridging integrator 1 (BIN1), also known as amphiphysin 2, is located on chromosome 2q14.3. As a member of the BAR family, BIN1 is involved in the regulation of the immune response, presynaptic neurotransmitter release, and the maintenance of calcium homeostasis (21). As one of the most stable genetic risk factors for DAT, BIN1 is overexpressed in patient brains (22). The level of neuronal BIN1 in the brain tissue of patients with DAT has been shown to be significantly lower than that of healthy people. In contrast, the level of ubiquitous BIN1 was shown to be significantly increased, as was the level of BIN1 in peripheral blood (23). This finding shows that different subtypes of BIN1 play a unique role in the occurrence and development of DAT. Rs7561528 is located 25 kB upstream of the BIN1 coding region. It has been reported that it affects a load of tau and the expression of BIN1 mRNA in the brain of patients with DAT (24). This effect is due to the endocytosis of BIN1 protein and its specific binding to tau protein, thereby limiting the extracellular pathological transmission of tau protein (25).

Another site of high BIN1 expression is skeletal muscle. A study found that BIN1 is the pathogenic gene of central nuclear myopathy (CNM). After the mutation of the BIN1 gene, the degradation of actin was shown to increase, and troponin was shown to be destroyed (26). In addition, BIN1 has been shown to aggravate the occurrence of progressive myasthenia through selective ectopic splicing and early specific invagination of T tubules in patients with ankylosis and malnutrition (27). The effect of high expression of BIN1 in the brain is not fully understood.

Therefore, we conducted a case-control study to detect and analyze the correlation between DAT and several polymorphisms in genes, including BIN1 and APOE, in the Chinese Han population.

## Materials and Methods

### Participants

The case-control study population included 137 patients with DAT (mean age ± SD = 72.77 ± 9.18; 56.20% female). Two trained psychiatrists made the DAT diagnoses. A senior associate professor of psychiatry clinically validated these diagnoses. Healthy controls (HCs) included a total of 509 cognitively healthy elderly individuals from the same geographic area as the patients (mean age ± SD = 65.78 ± 6.27; 51.08% female). Individuals free from any neurological, systemic, or psychiatric diseases and with no family history of neurological diseases were included in the HC group. The DAT group and HC group did not differ in terms of sex distribution, were composed entirely of Han Chinese individuals and included no blood relatives. The Mini-Mental State Examination (MMSE) and Clinical Dementia Rating (CDR) scale were performed for all participants. DAT patients were diagnosed according to the Diagnostic and Statistical Manual of Mental Disorders, Fifth Edition (DSM-V), criteria (28). According to the MMSE scale score, the selected participants in the HC group had the following characteristics: illiteracy >17 points, primary school >20 points, junior high school and above >22. The CDR scale score of HCs was 0. People under the age of 50 or with other neurological diseases, cancer, or a family history of autosomal-dominant dementia were not included in the study. People with a history of special drug usages and alcohol or drug dependence within the past 6 months were also excluded from this study. Participants were treated according to the ethical principles of the World Medical Association's Declaration of Helsinki. All participants signed the informed consent form, and the Institutional Ethical Committee approved the study. General demographic data, such as age, sex, education, occupation, and marital status, were collected.

### SNP Selection

First, we selected SNPs in the public HapMap database. The criteria for selecting SNPs were a minor allele frequency of (MAF) ≥0.05 and r2 ≥0.8 in the Han Chinese population of Beijing (HCB). A total of eighteen SNPs were selected for evaluation in this study: two SNPs of BIN1, one of RIN3, one of PICALM, one SNP of SPI1, one of TMP21, one of MTHFR, one of TMEM106B, one of MC1R, one of CENPO, one of PVRL2, one of KL, one of BZRAP1-AS, one of PFDN1/HBEGF, and three of APOE (**Table 2**).

### Genotyping

All participants were asked to fast for at least 8 h before morning blood collection. Approximately 5 ml of peripheral blood was collected from patients and controls, put into a test tube treated with EDTA and stored at 4°C. DNA was extracted from whole blood using a Tiangen DNA isolation kit (Tiangen Biotechnology, Beijing, China). TaqMan SNP genotyping analysis (Applied Biosystems, Foster City, CA, USA) and an ABI PRISM 7900 sequence detection system (Applied Biosystems) equipped with SDS2.1 software were used to analyze the selected SNPs of the genes. For quality control, researchers blinded to the status of the participant conducted the genotype analysis. Subsequently, 10% of the collected samples were genotyped again, and the results displayed 99.2% concordance. Two independent researchers scored the genotypic data. The deviation from the Hardy-Weinberg equilibrium expectation was evaluated for the entire study population.

### Statistical Analysis

A *t*-test was performed with SPSS 24.0 statistical software (SPSS Inc., Chicago, IL, USA) to evaluate the difference in age and education level between DAT patients and HCs. The Pearson chi-square test was used to determine differences in sex and allele and genotype frequencies between the two groups. Hardy-Weinberg equilibrium, allele frequency, and genotype frequency measurements were performed using SHEsis software (http://analysis.bio-x.cn/myAnalysis.php). Bonferroni's false discovery rate correction was applied. After adjusting for sex, education level, and age as possible influencing factors, SNPStats (https://www.snpstats.net/start.htm) was used to evaluate the association between SNPs and the risk of DAT under five inheritance models, including codominant, dominant, recessive, overdominant, and log-additive models. *P* <0.05 (two-tailed) was defined as a statistically significant difference.

## Results

### General Demographic Characteristics

In this study, 646 participants, including 137 patients with DAT and 509 unrelated HC individuals, were enrolled. In the DAT group, which included 78 females and 59 males, the mean age was 72.88 ± 9.11 years, ranging from 50 to 90 years. In the HC group, which included 260 females and 249 males, the mean age was 65.78 ± 6.72 years, ranging from 50 to 90 years. The two groups were not significantly different in terms of the sex distribution (*P* = 0.223, respectively). The mean education level of DAT patients was 8.46 ± 3.09 years, and the mean education level of the control participants was 10.29 ± 2.73 years. Thus, there was no difference in educational level between the two groups (Table 1).

**Table 1:** Characteristics of studied groups of persons.

	**DAT patients (*N* = 137)**	**Controls(*N* = 509)**	**χ2**	***P*-value**
Age, mean ± SD, years	72.77 ± 9.18	65.78 ± 6.27	-	<0.001^*^
Education, mean ± SD, years	8.50 ± 3.10	10.29 ± 2.73	-	0.068
Gender, female, *N* (%)	77 (56.20)	260 (51.08)	1.48	0.223

* *P <0.05*.

### Allele and Genotype Frequency of the SNPs

We detected and analyzed 18 DAT-related SNPs, including the BIN1 rs7561528 SNP. The allele frequencies and genotype frequencies of each gene polymorphism are listed in Table 2. The distribution of the APOE rs429358 and BIN1 rs7561528 polymorphisms in the population was in accordance with Hardy-Weinberg equilibrium. There were significant differences in the genotype frequencies (*P* = 0.001) and allele frequencies (*P* = 0.004) of rs429538 between the DAT group and the HC group. In the dominant model, after adjusting for age, sex, and education level, it was found that there was a significant difference in the APOE gene rs429538 polymorphism between the DAT group and the HC group (OR = 1.77, 95% CI = 1.04-3.00, *P* = 0.040). However, after Bonferroni's false discovery rate correction, the difference disappeared (adjusted *P* > 0.05, Table 3).

**Table 2:** Allele and genotype frequency of the SNPs.

**Closest gene**	**SNP**	***n***	**Allele n (%)**		**χ^2^**	***P***	**Minor Allele**	**Genotype n (%)**			**χ^2^**	***P***	***P* of HWE^a^**
BIN1	rs6733839		C	T	0.03	0.860	T	CC	CT	TT	1.26	0.530	
	DAT	86	94 (54.70)	78 (45.30)				30 (34.90)	34 (39.50)	22 (25.60)			0.060
	HC	460	496 (53.90)	424 (46.10)				142 (30.90)	212 (46.10)	106 (23.00)			0.120
BIN1	rs7561528		A	G	0.50	0.480	A	AA	AG	GG	3.26	0.200	
	DAT	118	29 (12.30)	207 (87.70)				4 (3.40)	21 (17.80)	93 (78.80)			0.060
	HC	495	139 (14.00)	851 (86.00)				9 (1.80)	121 (24.40)	365 (73.70)			0.780
RIN3	rs10498633		G	T	0.48	0.490	T	GG	GT	TT	2.15	0.340	
	DAT	135	247 (91.00)	23 (9.00)				112 (83.00)	23 (17.00)	0 (0.00)			0.280
	HC	509	917 (90.00)	101 (10.00)				416 (81.70)	85 (16.70)	8 (1.60)			0.140
PICALM	rs10792832		A	G	1.38	0.240	A	AA	AG	GG	5.31	0.070	
	DAT	129	96 (37.20)	162 (62.80)				23 (17.8)	50 (38.80)	56 (43.40)			0.050
	HC	507	418 (41.20)	596 (58.80)				83 (16.4)	252 (49.70)	172 (33.90)			0.560
SPI1	rs1057233		C	T	0.12	0.730	C	CC	TC	TT	0.25	0.880	
	DAT	93	52 (28.00)	134 (72.00)				7 (7.5)	38 (40.90)	48 (51.60)			0.890
	HC	471	275 (29.20)	667 (70.80)				43 (9.1)	189 (40.10)	239 (50.80)			0.520
TMP21	rs12435391		A	G	0.012	0.910	A	AA	AG	GG	0.05	0.980	
	DAT	133	45 (16.90)	221 (83.10)				4 (3.0)	37 (27.80)	92 (69.20)			0.900
	HC	503	173 (17.20)	833 (82.80)				17 (3.4)	139 (27.60)	347 (69.00)			0.510
MTHFR	rs1801133		C	T	0.55	0.460	T	CC	CT	TT	1.35	0.510	
	DAT	134	166 (61.90)	102 (38.10)				49 (36.60)	68 (50.70)	17 (12.70)			0.380
	HC	508	604 (59.40)	412 (40.60)				160 (31.50)	284 (55.90)	64 (12.60)			<0.01
TMEM106B	rs1990622		C	T	0.07	0.790	T	CC	CT	TT	1.12	0.57	
	DAT	134	181 (67.50)	87 (32.50)				63 (47.00)	55 (41.00)	16 (12.00)			0.460
	HC	504	672 (66.70)	336 (33.30)				220 (43.70)	232 (46.00)	52 (10.30)			0.420
MC1R	rs2228479		A	G	1.52	0.220	A	AA	AG	GG	1.54	0.460	
	DAT	135	51 (18.90)	219 (81.10)				5 (3.7)	41 (30.4)	89 (65.9)			0.920
	HC	503	225 (22.40)	781 (77.60)				25(5.0)	175 (34.80)	303 (60.20)			0.970
CENPO	rs6669072		C	T	0.48	0.490	T	CC	CT	TT	0.53	0.770	
	DAT	133	216 (81.20)	50 (18.80)				89 (66.90)	38 (28.60)	6 (4.50)			0.460
	HC	507	804 (79.30)	210 (20.70)				322 (63.50)	160 (31.60)	25 (4.90)			0.380
PVRL2	rs6859		A	G	1.11	0.290	A	AA	AG	GG	1.65	0.440	
	DAT	134	95 (35.40)	173 (64.60)				15 (11.10)	65 (48.50)	54 (40.30)			0.490
	HC	507	325 (32.10)	689 (67.90)				40 (7.90)	245 (48.30)	222 (43.80)			0.010
STARD6	rs10164112		C	T	2.00	0.160	T	CC	CT	TT	3.00	0.220	
	DAT	134	191 (71.30)	77 (28.70)				67 (50.00)	57 (42.50)	10 (7.50)			0.650
	HC	506	764 (75.50)	248 (24.50)				279 (55.10)	206(40.70)	21 (4.20)			0.020
APOE	rs7920721		A	G	0.006	0.940	G	AA	AG	GG	1.11	0.580	
	DAT	135	198 (73.30)	72 (26.70)				72 (53.30)	54 (40.00)	9 (6.70)			0.790
	HC	507	746 (73.60)	268 (26.40)				263 (51.90)	220 (43.40)	24 (4.70)			<0.01
APOE	rs429358		C	T	10.60	0.001^b^	C	CC	CT	TT	11.23	0.004^b^	
	DAT	116	40 (17.20)	192 (82.80)				3 (2.60)	34 (29.30)	79 (68.10)			0.770
	HC	478	93 (9.70)	863 (90.30)				3 (0.60)	87 (18.20)	388 (81.20)			0.430
APOE	rs7412		C	T	2.12	0.640	T	CC	CT	TT	1.98	0.370	
	DAT	131	248 (94.70)	14 (5.30)				118 (90.00)	12 (9.20)	1 (0.80)			0.280
	HC	484	890 (91.90)	78 (8.10)				418 (86.40)	54 (11.10)	12 (2.50)			<0.001
KL	rs9536314		G	T	0.00	1.000	G	GT	TT		0.00	1.000	
	DAT	310	0 (0.00)	268 (100.00)				0 (0.00)	134 (100.00)				1.000
	HC	310	2 (0.00)	1014 (100.00)				2(0.00)	506 (100.00)				1.000
BZRAP1-AS1	rs2632516		C	G	2.63	0.110	C	CC	CG	GG	3.31	0.190	
	DAT	134	113 (42.20)	155 (57.80)				21 (15.70)	71 (53.00)	42 (31.30)			0.320
	HC	505	482 (47.70)	528 (52.30)				115 (22.80)	252 (49.90)	138 (27.20)			1.000
PFDN1/HBEGF	rs11168036		G	T	0.16	0.690	T	GG	GT	TT	0.34	0.840	
	DAT	129	151 (58.50)	107 (41.50)				44 (34.10)	63 (48.80)	22 (17.10)			0.950
	HC	505	577 (57.10)	433 (42.80)				159 (31.50)	259 (51.30)	87 (17.20)			0.290

a *HWE, Hardy-Weinberg equilibrium test*.

b *After Bonferroni false discovery rate. correction, P <0.05*.

**Table 3:** Logistic regression analysis of SNPs.

**SNP**	**Inheritance model**		**OR (95%CI)**	***P* ^f^**
rs6733839	Codominant^a^	CC vs. CT	0.77 (0.43–1.39)	0.640
		CC vs. TT	0.97 (0.50–1.89)	
	Dominant^b^	CC vs. CT-TT	0.84 (0.49–1.43)	0.520
	Recessive^c^	CC-CT vs. TT	1.12 (0.62–2.01)	0.710
	Overdominant^d^	CC-TT vs. CT	0.78 (0.69–1.36)	0.350
	Log-additive^e^		0.97 (0.64–1.02)	0.850
rs10164112	Codominant	CC vs. CT	1.09 (0.69–1.72)	0.780
		CC vs. TT	1.37 (0.56–3.36)	
	Dominant	CC vs. CT-TT	1.12 (0.73–1.74)	0.600
	Recessive	CC-CT vs. TT	1.31 (0.55–3.16)	0.550
	Overdominant	CC-TT vs. CT	1.05 (0.68–1.64)	0.820
	Log-additive		1.13 (0.79–1.61)	0.500
rs10498633	Codominant	GG vs. TG	1.16 (0.64–2.09)	0.110
		GG vs. TT	0.00 (0.00–NA)	
	Dominant	GG vs. TG-TT	1.02 (0.57–1.83)	0.950
	Recessive	GG-TG vs. TT	0.00 (0.00–NA)	0.040
	Overdominant	GG-TT vs. TG	1.18 (0.65–2.13)	0.580
	Log-additive		0.90 (0.53–1.55)	0.710
rs10792832	Codominant	GG vs. AG	0.63 (0.39–1.04)	0.140
		GG vs. AA	0.98 (0.53–1.83)	
	Dominant	GG vs. AG-AA	0.72 (0.46–1.13)	0.160
	Recessive	GG-AG vs. AA	1.26 (0.72–2.22)	0.420
	Overdominant	GG-AA vs. AG	0.64 (0.41–1.00)	0.050
	Log-additive		0.92 (0.67–1.26)	0.590
rs11168036	Codominant	GG vs. TG	0.85 (0.52–1.39)	0.680
		GG vs. TT	0.76 (0.40–1.46)	
	Dominant	GG vs. TG-TT	0.82 (0.52–1.31)	0.420
	Recessive	GG-TG vs. TT	0.84 (0.47–1.51)	0.550
	Overdominant	GG-TT vs. TG	0.93 (0.60–1.45)	0.760
	Log-additive		0.87 (0.63–1.19)	0.380
rs12435391	Codominant	GG vs. AG	0.89 (0.55–1.46)	0.900
		GG vs. AA	0.94 (0.26–3.36)	
	Dominant	GG vs. AG-AA	0.90 (0.56–1.44)	0.650
	Recessive	GG-AG vs. AA	0.97 (0.28–3.45)	0.970
	Overdominant	GG-AA vs. AG	0.90 (0.55–1.46)	0.660
	Log-additive		0.92 (0.61–1.39)	0.690
rs1801133	Codominant	CC vs. CT	0.76 (0.47–1.23)	0.530
		CC vs. TT	0.83 (0.41–1.67)	
	Dominant	CC vs. CT-TT	0.77 (0.49–1.22)	0.270
	Recessive	CC-CT vs. TT	0.98 (0.52–1.86)	0.950
	Overdominant	CC-TT vs. CT	0.80 (0.52–1.24)	0.320
	Log-additive		0.87 (0.62–1.22)	0.410
rs1990622	Codominant	CC vs. CT	0.86 (0.54–1.36)	0.690
		CC vs. TT1.06	1.14 (0.56–2.32)	
	Dominant	CC vs. CT-TT	0.91 (0.56–1.41)	0.670
	Recessive	CC-CT vs. TT	1.23 (0.62–2.41)	0.560
	Overdominant	CC-TT vs. CT	0.84 (0.54–1.30)	0.430
	Log-additive		0.99 (0.71–1.38)	0.960
rs2228479	Codominant	GG vs. AG	0.77 (0.48–1.24)	0.560
		GG vs. AA	0.83 (0.28–2.45)	
	Dominant	GG vs. AG-AA	0.78 (0.50–1.23)	0.280
	Recessive	GG-AG vs. AA	0.91 (0.31–2.65)	0.860
	Overdominant	GG-AA vs. AG	0.78 (0.49–1.25)	0.300
	Log-additive		0.83 (0.56–1.22)	0.330
rs2632516	Codominant	GG vs. CG	0.83 (0.51–1.37)	0.170
		GG vs. CC	0.53 (0.27–1.04)	
	Dominant	GG vs. CG-CC	0.75 (0.46–1.20)	0.230
	Recessive	GG-CG vs. CC	0.60 (0.33–1.08)	0.080
	Overdominant	GG-CC vs. CG	1.06 (0.69–1.64)	0.800
	Log-additive		0.75 (0.54–1.03)	0.070
rs6669072	Codominant	CC vs. CT	0.96 (0.60-1.55)	0.970
		CC vs. TT	0.88 (0.30–2.58)	
	Dominant	CC vs. CT-TT	0.95 (0.60–1.50)	0.830
	Recessive	CC-CT vs. TT	0.89 (0.31–2.59)	0.830
	Overdominant	CC-TT vs. CT	0.97 (0.60–1.56)	0.900
	Log-additive		0.95 (0.65–1.39)	0.800
rs6859	Codominant	GG vs. AG	1.29 (0.81–2.05)	0.170
		GG vs. AA	2.06 (0.95–4.44)	
	Dominant	GG vs. AG-AA	1.39 (0.89–2.16)	0.150
	Recessive	GG-AG vs. AA	1.79 (0.89–2.16)	0.120
	Overdominant	GG-AA vs. AG	1.13 (0.73–1.74)	0.580
	Log-additive		1.38 (0.98–1.94)	0.070
rs7561528	Codominant	GG vs. AG	0.72 (0.40–1.29)	0.510
		GG vs. AA	0.75 (0.14–4.15)	
	Dominant	GG vs. AG-AA	0.72 (0.41–1.26)	0.250
	Recessive	GG-AG vs. AA	0.80 (0.15–4.42)	0.800
	Overdominant	GG-AA vs. AG	0.72 (0.40–1.29)	0.270
	Log-additive		0.76 (0.46–1.25)	0.270
rs7920721	Codominant	AA vs. AG	0.78 (0.50–1.23)	0.270
		AA vs. GG	1.61 (0.63–4.10)	
	Dominant	AA vs. AG-GG	0.85 (0.55–1.31)	0.450
	Recessive	AA-AG vs. GG	1.79 (0.72–4.48)	0.230
	Overdominant	AA-GG vs. AG	0.75 (0.48–1.16)	0.190
	Log-additive		0.97 (0.67–1.40)	0.850
rs9536314		TT vs. TG	0.00 (0.00–NA)	0.150
rs1057233	Codominant	TT vs. CT	0.99 (0.59–1.66)	1.000
		TT vs. CC	0.99 (0.40–2.43)	
	Dominant	TT vs. CT-CC	0.99 (0.60–1.62)	0.960
	Recessive	TT-CT vs. CC	0.99 (0.41–2.38)	0.990
	Overdominant	TT-CC vs. CT	0.99 (0.60–1.64)	0.970
	Log-additive		0.99 (0.68–1.45)	0.960
rs429358	Codominant	TT vs. CT	1.64 (0.95–2.83)	0.050
		TT vs. CC	5.41 (0.88–33.37)	
	Dominant	TT vs. CT-CC	1.77 (1.04–3.00)	0.040^g^
	Recessive	TT-CT vs. CC	4.82 (0.78–29.66)	0.090
	Overdominant	TT-CC vs. CT	1.58 (0.92–2.72)	0.100
	Log-additive		1.79 (1.11–2.89)	0.020^g^
rs7412	Codominant	CC vs. CT	0.82 (0.39–1.73)	0.270
		CC vs. TT	0.24 (0.03–2.01)	
	Dominant	CC vs. CT-TT	0.68 (0.34–1.39)	0.280
	Recessive	CC-CT vs. TT	0.25 (0.03–2.04)	0.130
	Overdominant	CC-TT vs. CT	0.84 (0.40–1.77)	0.650
	Log-additive		0.67 (0.37–1.21)	0.160

*CI, confidence interval; OR, odds ratio*.

a *Codominant, major allele homozygotes vs. heterozygotes*.

b *Dominant, major allele homozygotes vs. heterozygotes + minor allele homozygotes*.

c *Recessive, major allele homozygotes + heterozygotes vs. minor allele homozygotes*.

d *Overdominant, major allele homozygotes + minor allele homozygotes vs. heterozygotes*.

e *Log-additive, major allele homozygotes vs. heterozygotes vs. minor allele homozygotes. P-values were calculated by logistic regression analysis with adjustments for age, gender and education*.

f *The given P-values were not adjusted by Bonferroni correction*.

g *After Bonferroni false discovery rate correction, P > 0.05*.

Further stratified analysis was conducted with female and male subgroups, and the results are shown in Table 4. The results showed that the rs429538 C allele was a risk factor for DAT in females (*P* <0.001, OR = 2.83). The rs429538 CT and CC genotypes increased the risk of DAT by 2.64 and 14.3 times, respectively. The rs429538 polymorphism had no effect on DAT risk in men. The risk of DAT in men carrying the BIN1 rs7561528 A allele was significantly different from that in men carrying the BIN1 rs7561528 G allele (*P* = 0.021, OR = 0.33). Among men, the AG genotype was a protective factor for DAT (OR = 0.31). There was no significant difference in the risk of DAT among women with the BIN1 rs7561528 polymorphism.

**Table 4:** Association between SNPs and DAT risk according to the stratification by gender.

**SNP**	**Genotype**	**Male**				**Female**			
		N^*^/N	**B**	**OR (95%CI)**	***P***	N^*^/N	**B**	**OR (95%CI)**	***P***
APOErs429358	T	87/415	−1.41	-	-	107/448	−1.43	-	-
	C	13/53	−0.16	1.17 (0.61–2.24)	0.640	27/40	1.04	2.83 (1.66–4.81)	<0.001^*^
	TT	37/183	−1.60	-	-	43/205	−1.56	-	-
	CT	13/49	0.27	1.31 (0.65–2.66)	0.450	21/38	0.97	2.64 (1.41–4.93)	0.002^*^
	CC	0/2	−19.60	-	0.990	3/1	2.66	14.30 (1.45–140.80)	0.023^*^
BIN1rs7561528	G	95/416	−2.58	-	-	111/435	−1.37	-	-
	A	5/66	1.10	0.33 (0.130.85)	0.021^*^	23/73	0.21	1.24(0.74–2.06)	0.420
	GG	45/176	−1.36	-	-	48/189	−1.37	-	-
	AG	5/64	−1.19	0.31 (0.12–0.80)	0.016^*^	15/57	0.04	1.03 (0.54–1.99)	0.920
	AA	0/1	int	-	1.000	4/8	0.68	1.97 (0.57–6.81)	0.290

N^*^/N, DAT number of subjects/Control number of subjects. CI, confidence interval; OR, odds ratio.

* *P <0.05*.

### Association Between APOE Genotypes and DAT

The frequencies of apolipoprotein E genotypes and alleles were also analyzed (*P* <0.001; *P* = 0.001). The frequency of the ε4 allele in the DAT group (16.5%) was higher than that in the HC group (8.7%). The ε4 allele increased the risk of DAT by 2.78-fold (see Tables 5, 6).

**Table 5:** Association between APOE genotypes and DAT.

**Genotype**	**DAT**		**HC**		**χ2**	***P***
	***N***	**%**	***N***	**%**		
Genotypefrequency					28.45	<0.001^a^
E2/E2	1	0.9	6	1.3		
E2/E3	11	9.5	60	12.8		
E2/E4	0	0.0	2	0.4		
E3/E3	66	57.4	321	68.4		
E3/E4	34	29.6	80	17.1		
E4/E4	3	2.6	0.0	0.0		
Allelefrequency					13.45	0.001^a^
E2	13	5.3	74	7.9		
E3	190	78.2	782	83.4		
E4	40	16.5	82	8.7		

a *After Bonferroni false discovery rate correction, P <0.05*.

**Table 6:** Logistic regression analysis of APOE polymorphisms and DAT risk.

**Genotype**	**DAT**	**HC**	**B**	**OR (95%CI)**	***P***
Allele frequency					
E2	13 (5.3)	74 (7.9)	−1.74	-	-
E3	190 (78.2)	782 (83.4)	0.32	1.38 (0.75–2.55)	0.300
E4	40 (16.5)	82 (8.7)	1.02	2.78 (1.38–5.59)	0.004^*^

*CI, confidence interval; OR, odds ratio*.

* *P <0.05*.

## Discussion

Our study found that, in the dominant model, the APOE gene rs429358 polymorphism increased susceptibility to DAT. However, this difference disappeared after Bonferroni's false discovery rate correction. The rs429358 CC and CT genotypes were associated with an increased risk of DAT in women. The BIN1 rs7561528 polymorphism was associated with a decreased risk of DAT in men. These results suggest that there may be sex differences in the effect of APOE and BIN1 gene variations on DAT susceptibility.

Our results show that the ε4 allele of the APOE gene was overexpressed in DAT patients compared to HCs. Thus, considering ε2 gene carriers as a reference, the presence of the ε4 allele increased the risk of DAT. According to a genome-wide association (GWA) study, the APOE rs429358 polymorphism (APOE ε4 allele) is significantly associated with a reduced risk of longevity (29). In addition, the APOE rs429358 polymorphism is overexpressed in the brains of patients with PD and significantly affects cognitive dysfunction progression (30). Based on the analysis of gene polymorphisms of Hakkas in southern China, it was found that the risk of cerebral infarction in ε4 carriers was significantly higher than that in HCs (31). This finding suggests that ε4 may be a risk factor for longevity, PD, and cerebral infarction. Our research shows a significant increase in the risk of developing DAT among ε4 carriers, especially among women, consistent with the results of previous studies. Women who carry APOE ε4 have a much higher risk of developing DAT than men who carry APOE ε4. If a woman carries two ε4 alleles, her risk of developing DAT is 15 times higher than that of men who have the same genotype (32). Although the role of rs429358 in susceptibility to DAT in women is not fully understood, androgen may be a potential factor contributing to the increase in the prevalence of DAT. Recent studies have found that the decrease in androgen in DAT patients affects the APOE gene, leading to cognitive dysfunction and worsened development of DAT in these patients (33). A physiological dose of testosterone was suggested to be protective against on DAT. Androgen can alleviate the mitochondrial dysfunction caused by amyloid-beta (Aβ) and improve excessive oxidative stress in the brain tissue of patients with DAT (34). In addition, androgen is involved in the regulation of hippocampal formation and hippocampal-dependent cognitive behavior (35). Therefore, the high risk associated with the rs429358 polymorphism CC and CT genotypes may be related to the androgen deficiency in women.

In contrast, 65% to 80% of patients with DAT carry at least one copy of the APOE ε4 allele. Individuals with two APOE ε4 alleles had a 14 to 15-fold increased risk of DAT compared with those with no ε4 alleles (36). Mexicans who carry ε4 have a 13.3 times higher risk of developing dementia than those who are non-carriers (37). The inconsistency of risk results among different studies may be due to different races, environmental differences, and limited sample sizes.

Previous studies have suggested that APOE dysfunction causes disorders in maintaining cholesterol homeostasis in the brain by affecting cholesterol transport in the brain (37). This high-cholesterol state further promotes the formation of Aβ. It is well known that the accumulation of Aβ leads to the deposition of starch plaques in the terminal region of the cerebral cortex, which eventually leads to neurological degeneration and dementia by damaging nerve synapses (38). In addition, the different Aβ protein scavenging and proliferative functions of different subtypes of APOE may result in different risks of DAT among individuals carrying different APOE subtypes (39).

Rs7561528, the most significant SNP of the BIN1 gene, is considered a risk factor for late-onset Alzheimer's disease in the Caucasian population. The results of a large-scale meta-analysis suggest that rs7561528 A-allele carriers with the AG heterozygote genotype are not susceptible to DAT in any genetic inheritance pattern in mixed populations and East Asian populations. The AG heterozygote genotype of DAT is a protective factor for DAT in Caucasians and Asians (40). Unfortunately, this result has not been successfully replicated in the Han population in northern China (41). However, our study confirmed that male individuals with the A allele and AG genotype are not susceptible to DAT in the Chinese Han population.

Based on the analysis of other polymorphisms of the BIN1 gene in the Chinese Han population, rs67327804 and rs744373 were found to be risk factors for DAT (42, 43). These results suggest that mutations in the BIN1 gene are related to DAT in the Chinese Han population. A slight variation in BIN1 may lead to obvious pathological changes in individuals. For example, a BIN1 gene mutation can cause changes in internal olfactory structures and lead to hippocampal atrophy (44). However, variations in the BIN1 are not associated with the Aβ plaques observed in DAT. That is, the effect of BIN1 gene polymorphisms on DAT susceptibility may not be mediated by Aβ (45, 46).

There are several limitations to our study. First, our study had a cross-sectional design; therefore, the sequence of exposures, the timing of outcomes, and the causal relationship between exposures and outcomes were not considered. Second, although our sample size was significantly larger than those of previous reports, the limitations of our sample size, especially in the DAT group, resulted in our conclusions being less accurate or less persuasive. Therefore, these findings must be interpreted with caution. In the future, we will expand our sample size to further refine our conclusions. Third, our study did not take into account the linkage disequilibrium (LD) of SNPs. LD may affect the function of neighboring genes. Therefore, it is necessary to further understand the effect of LDs on samples. Fourth, since DAT is a multifactorial disease, other variables, such as marital, mental, and nutritional status, should be added to clinical data collection. Finally, although the Han people account for more than 90% of China's population, there are 56 ethnic groups in China; therefore, we should expand our research to different ethnic groups in the future.

## Conclusion

In conclusion, a variety of genes and their polymorphic genotypes are involved in the pathogenesis of DAT. Our study suggests that the APOE gene and BIN1 play an important role in the pathogenesis of DAT in the Chinese Han population. Sex differences also affect susceptibility to DAT. These results are of great significance for understanding the function and influence of different polymorphic genes in DAT. We believe that these results will help provide new insights into the diagnosis and treatment of DAT.

## Data Availability Statement

The original contributions presented in the study are included in the article/supplementary files, further inquiries can be directed to the corresponding authors.

## Ethics Statement

The studies involving human participants were reviewed and approved by Human Research and Ethics Committee of Wuxi Mental Health Center. The patients/participants provided their written informed consent to participate in this study.

## Author Contributions

KZ: conception and design. HL and KZ: administrative support. YZ: provision of study materials or patients. XC, HY, ZW, and GW: collection and assembly of data. YZ and XL: data analysis and interpretation. All authors manuscript writing and final approval of manuscript.

## Funding

We thank all of patients who volunteered to participate in the study. This study was supported by the National Natural Science Foundation of China (81801341), the Anhui Provincial Key R&D Programme (202004j07020030), the China International Medical Exchange Foundation (Z-2018-35-2002), and the National Clinical Key Specialty Project Foundation (CN). The funding body did not participate in the design, conduct, or writing of the study.

## Conflict of Interest

The authors declare that the research was conducted in the absence of any commercial or financial relationships that could be construed as a potential conflict of interest.

## Publisher's Note

All claims expressed in this article are solely those of the authors and do not necessarily represent those of their affiliated organizations, or those of the publisher, the editors and the reviewers. Any product that may be evaluated in this article, or claim that may be made by its manufacturer, is not guaranteed or endorsed by the publisher.
